# Beyond spring season: An autumn observation of adult lumpsuckers ( *Cyclopterus lumpus* ) in coastal Norway Frøya archipelago raises questions on extended spawning activity

**DOI:** 10.1111/jfb.70375

**Published:** 2026-02-24

**Authors:** Ole Henriksen, Mikael van Deurs, Rasmus Jacobsen, Thomas Warnar, Yvette Heimbrand

**Affiliations:** ^1^ Section for Ecosystem Based Marine Management National Institute of Aquatic Resources, Technical University of Denmark (DTU Aqua) Lyngby Denmark; ^2^ The Danish Society for Nature Conservation København Denmark; ^3^ Frederiksberg Gymnasium Frederiksberg Denmark; ^4^ Department of Aquatic Resources Swedish University of Agricultural Science Uppsala Sweden

**Keywords:** allochrony, lumpfish, phenology, reproduction, seasonality, spawning behaviour

## Abstract

During snorkelling near Frøya, central Norway, on 6 October 2025, a pair of adult lumpsuckers (*Cyclopterus lumpus*) was observed engaged in apparent prespawning behaviour in shallow kelp habitat. Such activity is surprising, as the species is mainly considered a spring spawner throughout the eastern Atlantic and is the focus of a targeted fishery during this period. Recent genomic work has identified a small, genetically distinct autumn‐spawning component in Norway. This observation provides rare behavioural support for that phenomenon, highlights its potential ecological relevance and proposes working hypothesis for future scientific studies.

The lumpsucker (also lumpfish, *Cyclopterus lumpus* Linnaeus, 1758) inhabits the cold and temperate North Atlantic from North American waters to Greenland and Iceland over the Barents Sea and southwards to the Greater North Sea and Baltic (Eriksen et al., 2014; Kennedy et al., 2015b). Population and genetic analyses reveal marked regional structuring with limited gene flow between oceanic sectors (Jansson et al., 2023; Jónsdóttir et al., 2018; Kennedy et al., 2015b; Kennedy, Post, Durif, et al., 2025). These are supported by studies that demonstrate long‐distance migrations between deep offshore feeding grounds and coastal spawning habitats (Mitamura et al., 2012; Kennedy & Ólafsson, 2019; Kennedy, Post, & Nøttestad, 2025). Despite this mobility, adults show homing behaviour and fine‐scale natal fidelity (Jansson et al., 2023; Kennedy et al., 2015b).

Historically, adults in the North Atlantic have been assumed to arrive at coastal spawning sites in spring when temperatures are rising after winter. The directed fishery exploits this spawning migration during spring and ceases by midsummer. Landings and effort data confirm that catches overwhelmingly consist of spring‐migrating females bearing ripe eggs, with no evidence of a corresponding autumn fishery (Kennedy et al., 2015a).

However, recent genomic and otolith shape analyses reveal greater temporal complexity. Horaud et al. (2025) identified parallel genomic divergence between spring and autumn spawners (temporal allochrony) along the Norwegian coast, particularly in Namdal and Sørøya, suggesting heritable adaptation to different seasonal regimes. The autumn group appears numerically small and geographically restricted, consistent with the historical absence of fisheries targeting it, but its existence demonstrates previously unrecognised flexibility in the species' reproductive timing.

Reproduction involves pronounced sexual dimorphism and male parental care. Males develop red or orange nuptial colouration and guard adhesive egg masses attached to stony habitat, kelp or even sand (Davenport & Thorsteinsson, 1989; Goulet et al., 1986; Goulet & Green, 1988).

On 6 October 2025, during a snorkelling excursion near Frøya, Trøndelag, Norway (63.7° N, 8.8° E; Figure 1), a pair of adult lumpsuckers was observed at ~2–4 m depth at a slope with dense *Laminaria hyperborea*. The male displayed bright orange‐red pigmentation and performed repeated circular and ‘blowing’ movements around a larger blue‐grey female attached to the kelp. This is a behaviour consistent with prespawning courtship (Goulet et al., 1986). The pair remained active and in close association throughout the observation of ~15–20 min, showing no apparent disturbance from the presence of the snorkelers (Figure 2a–d). All photographs and video material associated with this observation are available through DTU Data (DOI: https://doi.org/10.11583/DTU.30670634.v1).

**FIGURE 1:**
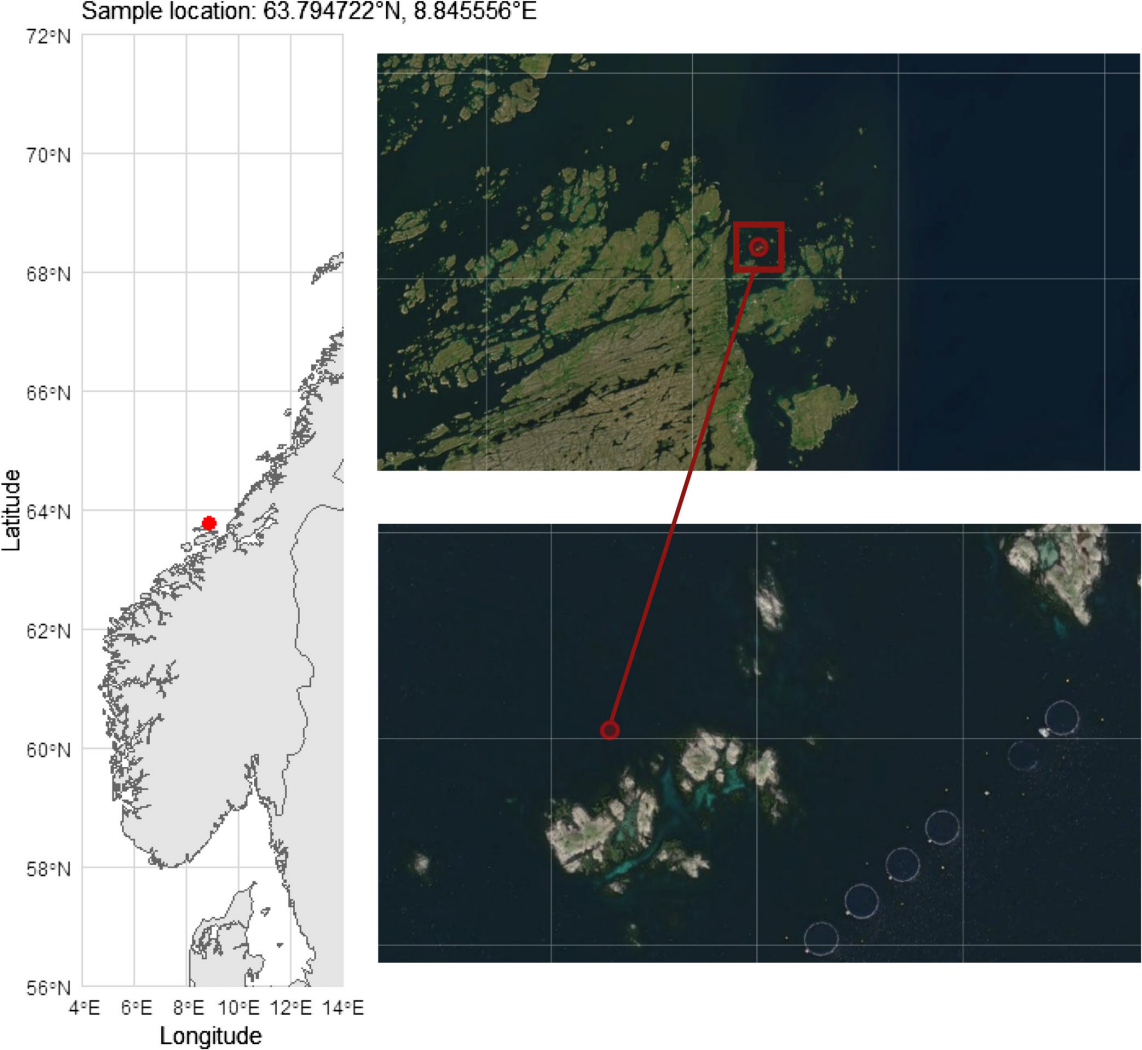
Maps showing the location of the *Cyclopterus lumpus* sighting near Frøya, Trøndelag, Norway. The zoomed maps show the archipelago (upper right panel) and the exact location near the rock island Ran with aquaculture cages in the background (lower right panel). The plot has been made using Create Interactive Web Maps with the JavaScript ‘*Leaflet*’ Library (Cheng et al., 2025).

**FIGURE 2:**
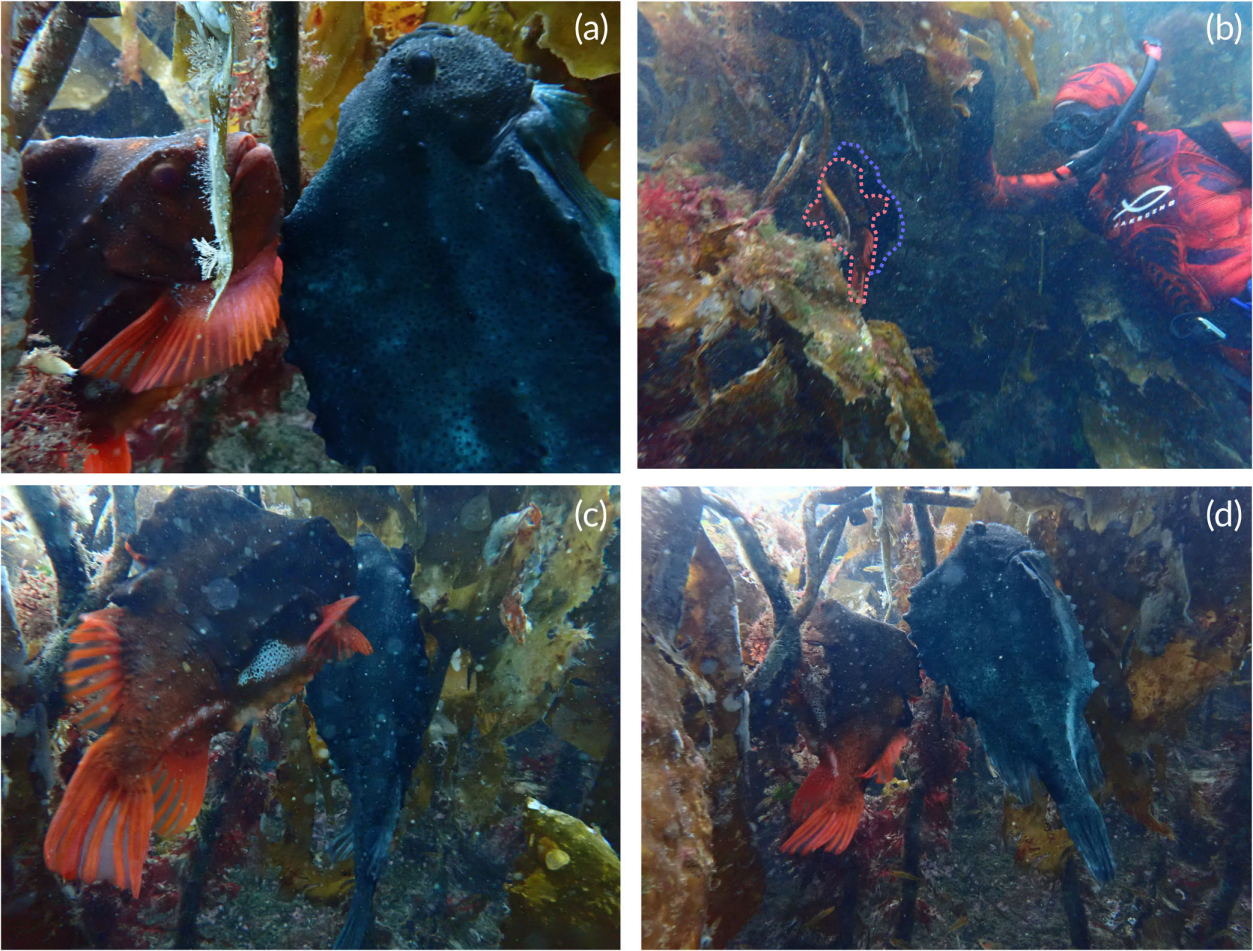
Pictures of the encounter. Close‐up of the brightly coloured orange‐red male positioned near a blue‐grey female, illustrating the male's characteristic nuptial colouration (a). The first and corresponding author observing the pair at close range, where both fish, male (annotated with red stippled line) and female (annotated with blue stippled line), appear undisturbed and fully engaged in courtship behaviour (b). Sequence showing the active circling displays a male around the stationary female, which remains attached by her pelvic disc to a frond of *Laminaria hyperborean* – note the angle of the pectoral and dorsal fins during display movements (c, d). Pictures were taken by coauthor Mikael van Deurs.

Although most lumpsuckers arrive inshore in spring, adults are occasionally seen as early as mid‐winter in northern areas (Durif, 2024). Whether the Frøya individuals represent early arrivals preceding the next spring season, late residual spawners or part of the autumn‐spawning lineage described by Horaud et al. (2025) is still unclear. Given the declining temperature and photoperiod at this latitude, it seems improbable that they were initiating the coming spring season. A more plausible explanation is that they were late‐maturing individuals originating from northern or offshore environments where cold conditions and short day length delay gonadal maturation or that they represent a distinct component of the population showing adaptions and temporal plasticity for autumn spawning.

This observation therefore adds behavioural context to the genetic evidence of Horaud et al. (2025) and demonstrates that reproductively active adults can occur several months after the presumed end of the spawning period. The lack of any historical fishery directed at autumn fish supports the view that such individuals form a small but potentially persistent temporal component of the population.

The occurrence of apparently reproductive lumpsuckers in October raises several non‐exclusive hypotheses:

**1) Photoperiodic and thermal plasticity:** Photoperiod exerts strong control over reproductive timing (Mlingi et al., 2024; Imsland et al., 2018a, 2018b). Laboratory manipulation can advance or delay spawning by months. Warming may decouple temperature and light cues, leading to asynchronous maturation or extended spawning seasons. Persistently mild autumns could promote delayed or secondary gonadal development, especially if spring conditions become suboptimal for spawning (Mortensen et al., 2020).

**2) Allochronic adaption:** Horaud et al. (2025) demonstrated genomic divergence between spring and autumn spawners in Norway, implying a stable evolutionary basis for dual spawning phases. The Frøya pair could belong to such an autumn lineage, extending its known range southwards. Even if numerically minor, these temporally separated groups could contribute disproportionately to resilience by spreading reproductive risk across seasons.

**3) Escapees or residual aquaculture fish:** Escapees from salmon farms can display atypical phenology (Powell et al., 2018), but the Frøya fish appeared behaviourally wild and although in close proximity to aquaculture facilities, we consider this explanation less likely.

## 1 CONCLUDING REMARKS

If late or autumn spawning increases under climate change, larval cohorts would encounter different environmental regimes, potentially altering survival and recruitment (Imsland et al., 2019). Conversely, phenological flexibility may buffer populations against mismatches between spawning and plankton availability. Recognising temporally distinct components, as identified by Horaud et al. (2025), will be critical for sustainable management.

Historically, fisheries have never targeted autumn spawners, implying that this component is both small and economically invisible. Yet its existence highlights that *C. lumpus* populations are structured not only spatially but also temporally. Further sampling of late‐season adults for gonadal staging, genetics and otolith microchemistry is needed to determine their contribution to recruitment and whether similar patterns occur elsewhere in the North Atlantic.

In conclusion, the Frøya observation provides field‐based support for multiple hypotheses proposed here. Nevertheless, recent genomic evidence for allochrony in lumpsuckers suggests that allochronic adaptation is the most plausible explanation. Yet, whether these late‐season fish are isolated anomalies, descendants of a distinct lineage or a climate‐driven extension of spawning activity remains an open question. Understanding such subtle temporal dynamics will be essential for predicting how this ecologically and economically important species responds to environmental change.

## AUTHOR CONTRIBUTIONS

This communication was conceived by all authors. The original manuscript was drafted by Ole Henriksen, and figure generation and edits were done by Ole Henriksen and Mikael van Deurs. All authors reviewed and edited the manuscript for accuracy.

## FUNDING INFORMATION

We also thank Project PRØV‐STEN funded by Fiskeafgiftsfonden for working hours drafting a manuscript, and Forskningsreserven for supporting and funding ongoing lumpsucker research and advisory efforts in Scandinavia.

## Data Availability

The data that support the findings of this study are openly available in DTU DATA at https://doi.org/10.11583/DTU.30670634.
